# Case Report: Proximal Phalangeal Fracture Management in a European Bison (*Bison bonasus*)

**DOI:** 10.3389/fvets.2022.859667

**Published:** 2022-04-04

**Authors:** Stefan Hoby, Adrian Steiner, Simone Jucker, Hansjürg Bähler, Maher Alsaaod

**Affiliations:** ^1^Berne Animal Park, Bern, Switzerland; ^2^Clinic for Ruminants, Vetsuisse Faculty, University of Bern, Bern, Switzerland

**Keywords:** European bison, fracture, proximal phalanx, lameness, radiographic evaluation

## Abstract

Fracture of the digits is a well-known orthopedic condition in adult cattle, and mainly the distal phalanx (P3) is involved. To our knowledge, the treatment of fractures of the middle (P2) and proximal (P1) phalanges with orthopedic claw blocks has not yet been described in cattle and other ruminants. This report describes the first case of the successful management of a P1 fracture in an adult European bison. A 5-year-old female European bison (*Bison bonasus*) presented with severe weight bearing lameness of the left hind limb and a marked soft tissue swelling accentuated over the plantar and lateral aspects of the proximal and middle phalanges, associated with multifocal light bluish discoloration of the skin (hematoma) and increased local temperature. The cow was examined and managed because of a severely comminuted fracture of the lateral P1 of the left hind limb. Application of an orthopedic block on the healthy medial partner claw allowed to reduce the load of the affected digit. Combined with the administration of non-steroidal anti-inflammatory drugs, it supported immobilization and recovery. Radiographic re-evaluations at weeks 6, 9, and 11 after the injury revealed progressive callus formation and fracture consolidation. From week 9 onwards, until the end of treatment, no lameness was observed. The healing process was good, and both clinical and radiographical improvement were evident after immobilizing the affected digit by applying an orthopedic claw block on the healthy partner claw and administering non-steroidal anti-inflammatory drugs.

## Introduction

Reports of phalangeal fracture management in wild ungulates are rare and limited to the distal phalanx (P3). A previous study by Rivas et al. (1) evaluated different fracture managements of P3 in two captive greater kudus (*Tragelaphus strepsiceros*) and one lesser kudu (*T. imberbis*) with overgrown front hooves. The latter was severely lame, and a block application was chosen as therapy method. The resolution of lameness was observed immediately after block application, and healing was confirmed radiographically at 8 weeks. Both greater kudus showed milder lameness and were managed conservatively by means of corrective hoof trimming, stall rest, and non-steroidal anti-inflammatory drugs (NSAIDs).

The fracture of the digits is a well-known orthopedic condition in adult cattle, and mainly P3 is involved (2). Severe trauma is the most common cause, and affected cattle show acute onset of signs of weight bearing lameness (3). Radiographic and/or ultrasonographic examination are critical in diagnosing the fracture (2, 4). In cattle, the usual treatment of phalangeal fractures consists of immobilizing the affected digit by applying an orthopedic claw block on the healthy partner claw of the affected limb (5) or using an external coaptation technique (6, 7). The orthopedic claw block must stay in place for 6–8 weeks, followed by radiographic reevaluation of the condition, block removal and block replacement if indicated. Radiographic assessment should always be complemented by clinical assessment to evaluate the healing process. To our knowledge, the treatment of fractures of the middle (P2) and proximal (P1) phalanges with orthopedic claw blocks has not yet been described neither in European bison nor in cattle.

The European bison (*Bison bonasus*) is the largest terrestrial European mammal. It has been extinct in the wild since 1927, but thanks to the breeding efforts of zoological institutions and reintroduction projects, the species was saved. In 2020, its conservation status was downgraded from “threatened” to “near threatened” according to the International Union for the Conservation of Nature (IUCN) Red List (8). This report describes the first case of the successful management of a P1 fracture in an adult European bison.

## Case Presentation

Berne Animal Park has a long tradition of keeping European bison, presenting their first offspring in 1958. Since 2009, the species is kept on a 49,000 m^2^ outdoor forest enclosure with natural floor. In 2020, a squeeze chute designed for the American bison (*Bison bison*) with an integrated scale (Pearson, Vernon, USA) was installed. It was adapted with the aim of investigating the foot health within the framework of an ongoing animal experiment (license no. BE 10/2021) as a result of digital dermatitis associated with *Treponema* spp. (9). A 5-year-old female European bison with a body weight of 420 kg was trained during several weeks to access the squeeze chute. After the animal was physically restrained in the chute, we attempted to lift a hind limb to examine the foot, analogous to cattle. At this very moment, the cow vehemently kicked out with the left hind limb to the rear, thereby hitting a metal bar of the chute. Thereafter, the European bison hardly bore its weight upon the left hind limb in the chute. The chute was opened, and severe lameness [score 5/5 according to Sprecher et al. (10)] during the weight bearing phase of the stride was evident and did not improve even after prolonged treatment with NSAIDs. Initially, carprofen (Rimadyl Rind, Zoetis, Switzerland, 1.4 mg/kg once) followed by meloxicam (Metacam 20 mg/ml, Boehringer, Switzerland, 0.5 mg/kg SID q24 h 2 days) was administered i.m. by dart gun (Daninject, Gelsenkirchen, Germany). Thereafter, ketoprofen (Dolovet, Graeub, Switzerland, 4.5 mg/kg SID 8 days) was administered p.o. There was no visible axial deviation of the limb or obvious skin lesion, but a soft tissue swelling extending from the lateral fetlock to the pastern joint was evident.

**Figure 1:**
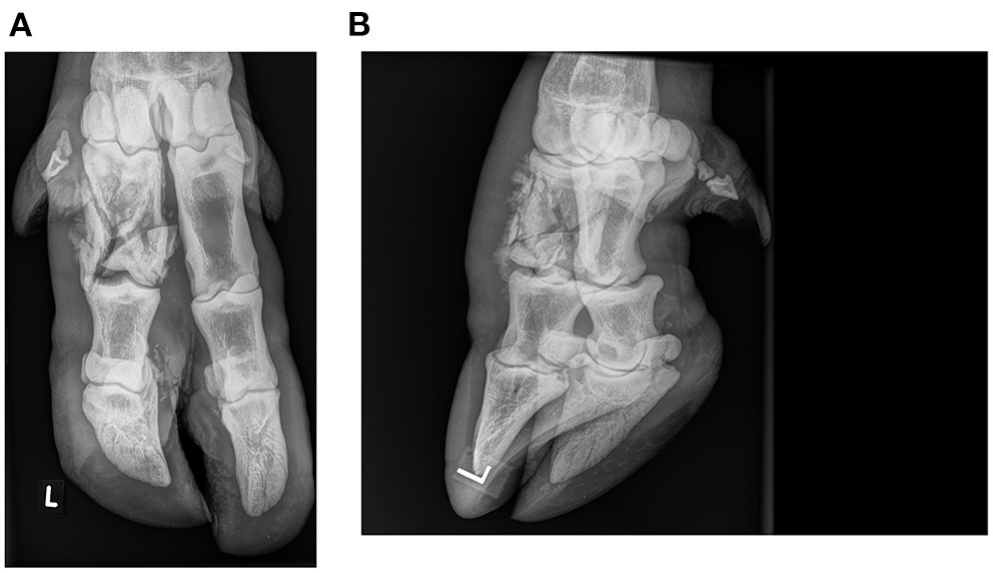
Plantarodorsal **(A)** and oblique lateral **(B)** radiographs of a multifragmentary fracture of the lateral proximal phalanx in an adult female European bison (*Bison bonasus*): One month after the injury and immediately before applying an orthopedic claw block.

Due to persistent lameness (scores 4/5–5/5) for 4 weeks, the animal was anesthetized after separation in a box and overnight fasting. Medetomidine (Medetomidin HCL, Christoffel Apotheke, Bern, Switzerland, 0.08 mg/kg) and ketamine (Ketamin HCL, Christoffel Apotheke, Bern, Switzerland, 2.5 mg /kg) were applied in a mixed syringe by dart gun in the shoulder musculature. The animal was placed in right lateral recumbency, received 4.2 liter of oxygen/min per nasal tube and meloxicam (Metacam® 20 mg/ml, Boehringer, Switzerland, 0.5 mg/kg, i.m.). Additional medication included subcutaneous administration of 375.0 mg vitamin E and 15.0 mg sodium selenit (Tocoselenit, Graeub, Switzerland) and 1.0 mg of vitamin B12 (Catosal, Covetrus, Switzerland). To reverse anesthesia, atipamezole (Alzane, Graeub, Switzerland, 0.4 mg/kg bw, i.m.) was administered. The claws and the interdigital space of the left hind limb were cleaned routinely. Thereafter, the hair of the foot from the coronary band up to the fetlock joint was clipped. Radiographs were taken with a portable generator (SP-Vet-4.0, Sedecal, Spain) and associated digital radiography cassette system (Agfa DR 14e, Schweizer, Switzerland). Exposure indices were 63 kV and 5 mAs. During the procedure, the cassette was kept in a protective case with a removable handle (DR-Cowboy, Podoblock, Tynaarlo, the Netherlands). A plantaro-dorsal and an oblique lateral radiograph were made, adapted from cattle (11). Upon clinical examination, a marked soft tissue swelling, mainly on the plantar and abaxial aspect of P1 and P2 was evident, associated with multifocal light bluish discoloration of the skin (hematoma) and increased local temperature. Radiographic evaluation revealed a closed, complete, severely comminuted articular fracture of the left hind lateral P1 (Figures 1A,B) (12). The multifragmentary fracture that involved the proximal interphalangeal joint showed signs of mild callus formation on the axial and abaxial aspects of P1. Of the three main fragments, the two distal ones were moderately displaced in proximo-axial and proximo-abaxial direction, respectively. Marked soft tissue swelling extended from the fetlock to the pastern joint. Rectal examination revealed pregnancy in the last trimester.

**Figure 2:**
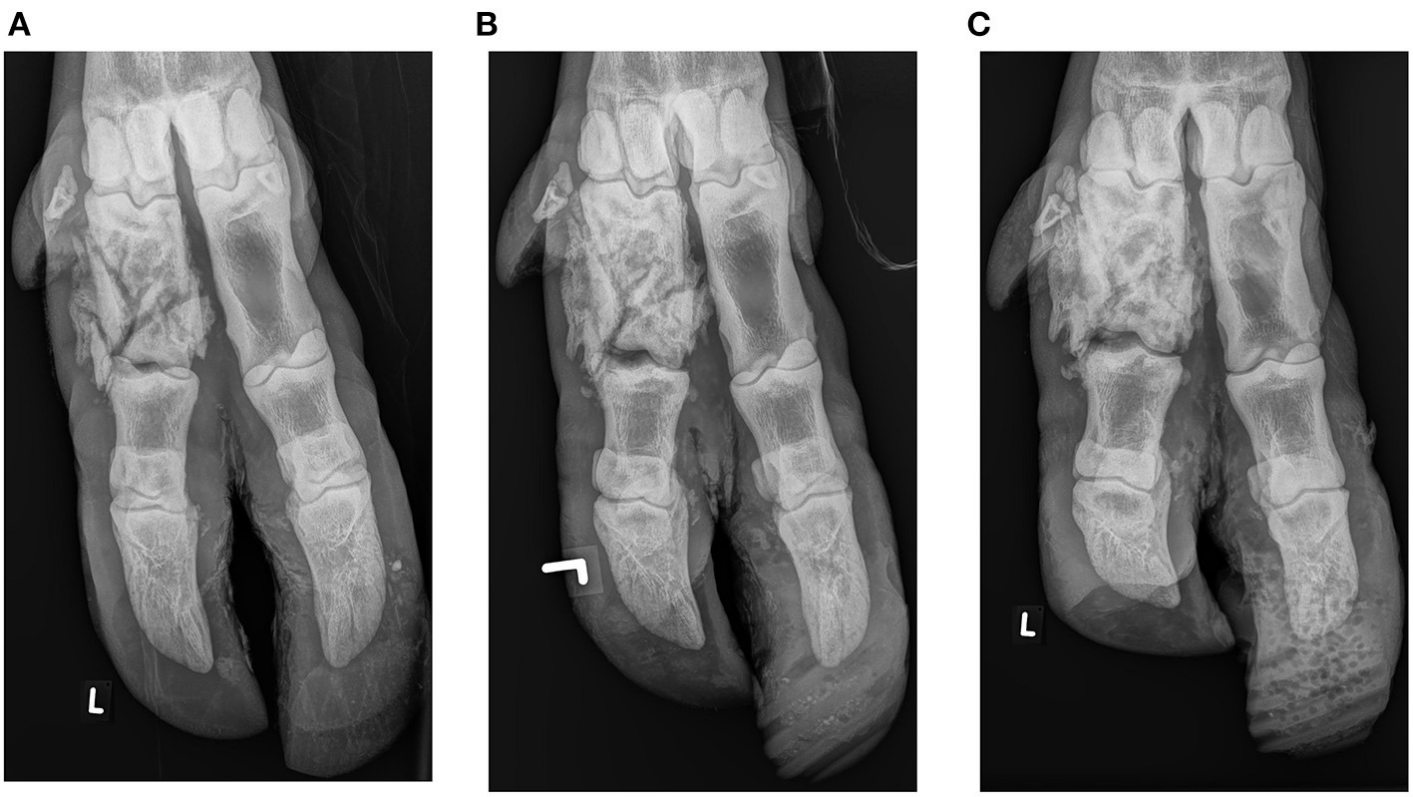
Plantarodorsal radiographs of a multifragmentary fracture of the lateral proximal phalanx in an adult female European bison (*Bison bonasus*): Six **(A)**, 9 **(B)**, and 11 weeks **(C)** after the injury. At the initital treatment, a wooden claw block was used and polyurethane blocks thereafter.

**Figure 3:**
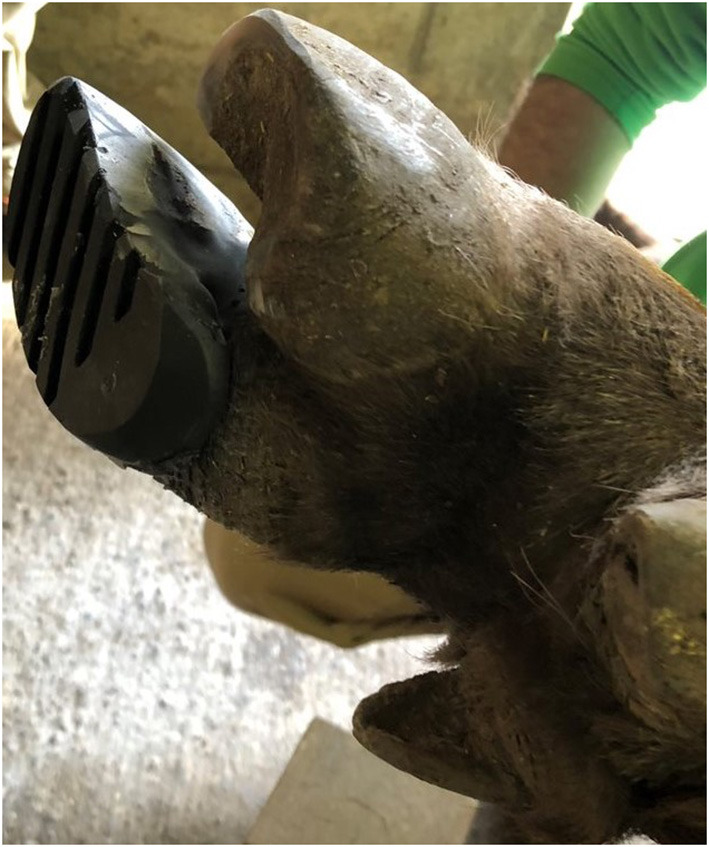
Orthopedic polyurethane claw block fixed to the healthy partner claw for treatment of a multifragmentary fracture of the lateral proximal phalanx of the left hind limb of an adult female European bison (*Bison bonasus*).

Initial local treatment consisted of application of an orthopedic wooden block of 2.5 cm thickness on the medial claw of the left hind limb, using polymethlymethacrylate adhesive (Technovit, Covetrus, Switzerland). The block was shaped to the contour of the specific claw, and the curing time of the adhesive was shortened by using a hot air dryer. Special care was taken not to apply adhesive to the soft horn of the heel. The lameness was rated 3/5 after initial local intervention. Block replacement was performed after 6, 9 and 11 weeks (Figures 2A–C). After the second replacement, the lameness score of 2/5 remained until the end of treatment. Due to the heavy wear of the wooden block after only 2 weeks in place, a polyurethane block (MS Schippers, Hapert, the Netherlands) with a thickness of 2.5 cm was used for the subsequent three treatments (Figure 3). The same adhesive as described above was used. Control radiographs were taken at weeks 6, 9, and 11 after the injury. At week 9, the block was judged stable and with minimal wear, so it was not changed. However, 2 days later it fell off. Signs of lameness were not present at that time. Nevertheless, the block was replaced the next day. Radiographic evaluations were performed at 4, 6, 9, and 11 weeks after the injury and revealed progressive callus formation, consolidation of the fracture lines, bone remodeling and continuously decreasing soft tissue swelling without further displacement of fracture fragments (Figures 2A–C). Radiographic signs of ankylosis of the proximal interphalangeal joint were not evident. This was consistent with clinical improvement evidenced by progressive gait improvement.

At week 16, the animal gave birth to a healthy calf and presented in good overall condition without lameness. The block, attached at week 11, was still in place. Due to the physiological position of the foot, the presence of the newborn calf and the increasing wear of the block, the block was not removed. It fell off at week 26, and the cow remained non-lame.

## Discussion

We describe for the first time the successful treatment of a multifragmentary P1 fracture in a European bison. Application of a block to the healthy partner claw is a non-invasive technique and reduces pain during walking (13), allows for passive fracture immobilization and therefore supports fracture healing. Its use in treating P3 fractures in cattle is common and well-described (14), but reports in wild ungulates are very limited (1, 15). Open reduction and internal fixation was initially discussed as a treatment option due to the involvement of the proximal interphalangeal joint. This option was discarded due to the multifragmentary nature of the fracture and the expected long duration of general anesthesia required. Additionally, an external coaptation technique with stall rest that was successfully applied in cattle was deemed inappropriate for a wild European bison (6).

Due to the minimal mechanical abrasion, we recommend using polyurethane orthopedic blocks instead of the commonly applied wooden blocks for European bison in the field. In this study, NSAIDs were repeatedly administered, starting immediately after the injury whereas the orthopedic claw block was applied 4 weeks later. In previous studies in cattle, it was shown that applying an orthopedic claw block significantly improved weight bearing while walking but not during standing, and administration of the NSAID ketoprofen improved weight bearing during walking as well as standing (13, 16). Radiographic and/or ultrasonographic examination are critical in diagnosing the fracture. While radiographic examination is essential to assess the fracture, ultrasonographic examination may provide earlier evidence of the healing process of a fractured phalanx (17).

## Conclusion

Based on the successful outcome of the case described here, we recommend the claw block application on the partner claw and systemic administration of NSAIDs for treatment of proximal phalangeal fractures, in order to promote rapid and undisturbed recovery and improve the wellbeing of the animal. However, we discourage the incautious confinement of European bison for the sake of inspection of the feet. Individual temper, character and training level of the animal should always be traded against the potential threats such as severe injuries. At the very least, using sedatives before foot manipulation should be taken seriously into consideration.

## Data Availability Statement

The original contributions presented in the study are included in the article/supplementary material, further inquiries can be directed to the corresponding author/s.

## Ethics Statement

The animal study was reviewed and approved within the framework of an ongoing animal experiment (license no. BE 10/2021). Written informed consent was obtained from the owners for the participation of their animals in this study.

## Author Contributions

SH was responsible for data collection and writing the first draft of the manuscript. SJ and HB supported the data collection. AS and MA edited the manuscript and supervised the study. All authors contributed to the manuscript and approved the final version.

## Conflict of Interest

The authors declare that the research was conducted in the absence of any commercial or financial relationships that could be construed as a potential conflict of interest.

## Publisher's Note

All claims expressed in this article are solely those of the authors and do not necessarily represent those of their affiliated organizations, or those of the publisher, the editors and the reviewers. Any product that may be evaluated in this article, or claim that may be made by its manufacturer, is not guaranteed or endorsed by the publisher.
